# Relationship between the animal body condition and reproduction: the biotechnological aspects

**DOI:** 10.5194/aab-63-203-2020

**Published:** 2020-07-01

**Authors:** Jiří Bezdíček, Andrea Nesvadbová, Alexander Makarevich, Elena Kubovičová

**Affiliations:** 1 Faculty of Science, Palacký University Olomouc, Olomouc, 771 47, Czech Republic; 2 National Agricultural and Food Centre (NPPC), Research Institute for Animal Production Nitra, 95141 Lužianky, Slovak Republic

## Abstract

The aim of this review was to evaluate the relationship between the body condition of cows and reproduction. Reproduction was evaluated from the viewpoint of animal husbandry traits, ovarian activity and embryo transfer. Main emphasis was given to the review of articles from the area of biotechnical methods (in vitro embryo production, embryo transfer). Most authors agree on the opinion that the worsening of the reproduction traits of cows is a result of changes in the body condition score (BCS) either under or over their average value. Worsening of reproduction traits was presented not only from a zootechnical viewpoint (e.g., calving interval, 56 d nonreturn rate, etc.) but also in term of ovarian activity, oocyte recovery and in vitro embryo production. In general, the body condition of cows is an important factor affecting female reproduction ability at the ovarian level.

## 1 Introduction

Animal body condition is a crucial factor closely related to many traits, such as reproduction, health, production (e.g., milk) and others. In general, when animals live in a suitable environment, the body condition does not change significantly during the year. A specific situation occurs in dairy cows with various intensity of milk production during lactation, which is reflected in different energy demands. Thus, the body condition of dairy cows can change considerably during one calving interval (period between two births; lactation plus dry period). High energy demands occur in cows during the postpartum period, when milk production intensifies and body fat and energy reserves drop. This period of high milk production is also associated with forthcoming pregnancy. A decrease in the body condition score (BCS) during the postpartum period has been documented by several authors (Roche et al., 2007; Koenen et al., 2001; Friggens et al., 2004). Particularly, Bastin et al. (2010) proved a decrease in BCS during the first part of lactation in Canadian Holstein and Ayrshire cows and the lowest BCS for both breeds after about 60 d in milk. Buckley et al. (2003) also reported that in the period between calving and first service the body condition score declined dramatically. At the same time, these authors state that the BCS should not fall by more than 0.5 units to avoid a negative effect on reproduction traits. In this regard, the authors also found that a substantial reduction in the body condition is associated with a lower pregnancy rate after 42 d (Buckley et al., 2003).

Research on the relationship between the body condition and reproduction has been carried out by a number of research teams and from different viewpoints. Important studies have been focused on animal husbandry (Buckley et al., 2003; Berry et al., 2003; Banos et al., 2007; Cam et al., 2018; Gruber et al., 2018; Makarevich et al., 2018; Ledinek et al., 2019; Ptáček et al., 2017; Tančin et al., 2018), but also on biotechnology, e.g., embryo transfer (Bezdíček et al., 2015; Bezdíček and Louda, 2016), in vitro fertilization (Makarevich et al., 2012b; Chrenek et al., 2015; Oba et al., 2013) and ovarian activity (Makarevich et al., 2012a; Kuźnicka et al., 2016). A number of studies have been also focused on the relationship between the body condition and reproduction from a veterinary point of view, e.g., early embryonic mortality and retained placenta (Qu et al., 2014; Aungier et al., 2014; Berry et al., 2007a, b). A relationship was also determined between BCS and mastitis by Loker et al. (2012), who calculated the average genetic correlation between the BCS and mastitis in Canadian Holstein (-0.730) and the correlation between BCS and metabolic disease (-0.438). These authors reported a higher susceptibility to mastitis in cows with a lower BCS (Loker et al., 2012).

The worsening of the body condition is thus associated with a number of important breeding aspects. The aim of this review is to evaluate the relationship between the body condition and reproduction in cows from different viewpoints: (1) animal husbandry, (2) ovarian activity and the subsequent production of oocytes and embryos, and (3) embryo transfer in dairy cows. The greatest attention is devoted to ovarian activity.

## 2 The system of body condition score (BCS)

At present, an evaluation of a cow's body condition is carried out very effectively using the body condition score (BCS) system based on a five-point (or different, e.g., nine-point) scale. This system allows a subjective evaluation of the body condition and also a subsequent statistical evaluation of the obtained values. For example, in the case of a five-point scale in Holstein dairy cows, score 1 means a severe under-condition, 3 means an optimal condition and 5 a severe over-condition (Wildman et al., 1982; Edmonson et al., 1989; Ferguson et al., 1994). This evaluation is subjective, and therefore the experience of evaluators plays an important role. Thus, there are efforts to develop an objective system of BCS evaluation (Spoliansky et al., 2016; Azzaro et al., 2011; Ferguson et al., 2006). These systems use different types of cameras, not only digital (Bercovich et al., 2013; Bewley et al., 2008) but also thermal cameras (Halachmi et al., 2008).

Currently, the objective evaluation systems are significantly advanced from 2D to 3D (three-dimensional) visualization, allowing for monitoring the animal contours as well (Weber et al., 2014; Shelley et al., 2016; Hansen et al., 2018; Shigeta et al., 2018). There are numerous three-dimensional systems nowadays, but the important thing is to consider their applicability to different cattle breeds. According to Alvarez et al. (2018) it would be appropriate to analyze these parameters specifically for each cattle breed.

## 3 Body condition of cows from the viewpoint of animal husbandry

Generally, reproduction in cows is evaluated from different viewpoints. Animal husbandry observes the following traits: age at first calving, days between first service and conception, 56 d nonreturn rate at first insemination, length of calving interval, number of inseminations per lactation, and other reproduction indicators that have been analyzed in a number of studies in the past years in relation to various influences, including body condition.

These animal husbandry indicators (traits) are very important and understandable in practice, but they are generally influenced by a number of external or internal factors. For this reason, reproduction research has currently moved into the field of gametes, ovarian activity and embryo production where reproduction begins. Research at this level has enabled the development of biotechnical methods in cattle breeding, especially embryo transfer, in vitro fertilization and embryo culture, as well as technical equipment, e.g., sonography and micromanipulation with gametes.

Most authors agree on the negative genetic correlation between reproduction and BCS in animal husbandry traits. In particular, Bastin et al. (2010) reported negative genetic correlations between BCS and days from calving to first service, days from first service to conception, and open days. For Holstein cows these correlations ranged between -0.31 and -0.03. Positive correlations were found between BCS and a 56 d nonreturn rate at first insemination (Bastin et al., 2010). Based on these results, the authors concluded that in Holstein and Ayrshire cows, a genetically low BCS at early lactation is connected with a longer nonpregnant time and a lower chance of pregnancy after the first service. Similarly, Berry et al. (2003) presented a genetic correlation between BCS and the interval to first services that ranged from -0.47 to -0.31. The correlation between BCS and fertility has been determined in a number of studies (Chebel et al., 2018; Pryce et al., 2001, 2002; Dechow et al., 2001; 2002; Gillund et al., 2001; Domecq et al., 1997; Banos et al., 2007). Many studies also emphasize the nonlinearity of these relationships (Tiezzi et al., 2013).

## 4 Body condition of cows from the viewpoint of embryo transfer

In the past years, embryo transfer has become a mastered biotechnology method in cattle. This biotechnique has enriched the research with very important knowledge about a number of factors affecting animal reproduction. These are factors such as temperature stress, the effect of an individual (due to the large number of offspring) and the effect of the lactation stage. The results of embryo production (embryo transfer) were also evaluated in terms of animal body condition.

In this field, Bezdíček et al. (2015) reported that better results in embryo transfer were achieved in Holstein cows with an optimal body condition (BCS 3.0–3.5) than in cows with a tendency to obesity or emaciation. Specifically, the animals divided into three groups according to their body condition (BCS less than 2.9, 3.0–3.5 and above 3.5 points) produced an average number of flushed embryos per flushing as follows: 3.4 (±0.690), 4.6 (±0.917) and 1.9 (±1.659) pieces, respectively. Also, in terms of embryo quality (transferable or degenerated) better results were obtained in cows with an optimal body condition. In the studied BCS groups the following results for the number of transferable (pieces) and the proportion of degenerated embryos (%) were obtained: BCS up to 2.9 (2.58 pieces and 31.05 %), BCS 3.0–3.5 (3.41 pieces and 28.31 %) and BCS over 3.5 (1.66 pieces and 19.07 %), respectively. However, the differences among the groups were statistically insignificant (Bezdíček et al., 2015).

Relationships between fertility and changes in body weight (BCS) were also the subject of the study by Carvalho et al. (2014). The authors divided the cows at the postpartum period into four quartiles (Q) that presented changes (%) in body weight (Q1 indicates the smallest change; Q4 indicates the biggest change). Comparison of groups Q1 and Q4 revealed a higher proportion of transferable embryos (83.8 % vs. 53.2 %) and a lower proportion (9.6 % vs. 35.2 %) and count (0.6 % vs. 2.7 %) of degenerated embryos in the Q1 group (smallest change). In conclusion, the authors concluded that these results are in compliance with the idea that a lower BCS at the time of the first insemination is associated with fertility decline (Carvalho et al., 2014).

The relationship between embryonic losses in dairy cows was studied by Silke et al. (2002). The authors state that changes in BCS in dairy cows are connected with embryonic mortality. It was found that a higher rate of embryonic loss (11.6 %) is associated with a decline in BCS during 28–56 gestation days. With a change in the body condition by -1 point, the regression of embryo loss was 0.211; in the case of an unchanged body condition or change by +1 point, the probability of embryo loss in cows was 0.065 or 0.018 (Silke et al., 2002).

Kadokawa et al. (2008) studied the relationship between BCS and embryo production in Holstein heifers (Iwate Station, Japan). In this study concentrations of insulin and glucose were also determined. The results showed that heifers with BCS 3.5 showed a lower count of flushed excellent embryos and total number of embryos than heifers with BCS 3.00 or 3.25. Heifers with BCS 2.75 tended (p=0.06) to produce more unfertilized oocytes than heifers with the body condition score 3.25. The results also showed that heifers with BCS 3.5 were hyperinsulinemic (blood insulin concentration was 155.8±14.0 pM), while in heifers with BCS 2.75 the insulin concentration was 125.6±15.1 pM (Kadokawa et al., 2008).

In general, cows (or heifers) with excellent results in milk production are used for embryo transfer. This technique is mostly carried out at the best farms, and therefore it is highly unlikely to find animals with a poor body condition at extreme values (BCS 1 and 5). In principle, these animals are not used for embryo transfer. Nevertheless, in practice there is an obvious tendency and recommendation to use animals at an optimal body condition (BCS 3) for embryo transfer.

In recent years, research in this area has focused not only on monitoring the number of obtained embryos or their quality, but also on ovarian activity, such as the number of corpus luteum, the size of the dominant follicle and the size of the ovary. Thus, research on body condition influence is focused on ovarian activity (Boland et al., 2001; Makarevich et al., 2011; Sirotkin et al., 2013).

## 5 Body condition of cows from the viewpoint of ovarian activity, oocyte recovery and embryo production in vitro

A healthy and functional corpus luteum is very important for optimal reproduction. A number of research papers in this field demonstrate that animals with a poor body condition (thin or fat cows) evidence changes in histopathological images of corpus luteum. This is associated with decreased progesterone production. In particular, Makarevich et al. (2012a) found that the average number of active corpora lutea (determined postmortem) was lower in animals with a lower body condition (BCS 1) than in animals with an optimal body condition (BCS 3). This also corresponded to the number of detected corpus albicans. Specifically, the numbers of corpus luteum in BCS 1 vs. BCS 3 were 0.19 vs. 0.37 pieces, respectively; in the case of corpus albicans it was 0.25 vs. 0.38 pieces, respectively. These differences were statistically significant. These authors also reported that no significant differences in the mean ovary area between cows of different BCS groups (BCS 1, BCS 3, BCS 4: 9.40, 7.74, 8.14 cm${}^{2}$) were observed (Makarevich et al., 2012a). The size of the ovarian area was also studied by Sirotkin et al. (2013), who reported a smaller ovarian area in emaciated cows (BCS 2) compared to cows with the optimal body condition (BCS 3).

Murphy et al. (1991) found that low dietary intake in beef heifers is associated with a smaller diameter of dominant follicles during the oestrous cycle. The maximum diameter of dominant follicles was significantly smaller (11.8±0.1 mm) in the case of low diet (feeding 0.7 % of body weight in dry matter) in comparison with the heifers fed 1.1 % or 1.8 % of their body weight in dry matter. In this case the diameter of dominant follicles was 13.7±0.2 or 13.2±0.3 mm, respectively (Murphy et al., 1991).

Beam and Butler (1997) also found that higher fat intake is linked to an increased diameter of dominant follicles. The authors present evidence that cows with a moderate-fat or high-fat diet generate a greater number of follicles (>15 mm) compared to cows with a low-fat diet.

A poor body condition (mainly BCS 1) in cows may even result in the malfunction of ovarian activity in the form of cystic atresia of ovarian follicles (Pivko et al., 2012). Thus, the ratio of cystic atresia increased in the case of emaciated cows (BCS 1 and 2) in comparison to cows with an optimal body condition (BCS 3) (Makarevich et al., 2011; Pivko et al., 2012). The authors in these studies also report that in cows with a very poor body condition (BCS 1) the percentage of ovulated follicles was lower (19.0 %) than in cows with BCS 2 (76.6 %) or BCS 3 (68.7 %). Sirotkin et al. (2013) documented that in cows with a tendency to emaciation (BCS 2) ovarian function may be suppressed by the increased production of the oestradiol hormone.

Kubovičová et al. (2012), using ovaries from 163 cows slaughtered at the abattoir, studied the influence of the animal body condition on the number of follicles on the ovary. The results showed that animals with an optimal body condition (BCS 3) had a significantly higher number of follicles per ovary than animals tending to fattening (BCS 4) or an emaciated condition (BCS 1). For each group (BCS 1, BCS 3, BCS 4) the follicle counts were as follows: 13.33±1.31, 18.04±2.66 and 5.19±1.24 pieces, respectively (Kubovičová et al., 2012). Statistically significant differences were found between BCS 3 and BCS 4. Also, in Polish Friesian cows, Nowak et al. (2011) documented a lower mean number of follicles and aspirated oocytes in extremely emaciated cows.

The relationship of body condition to the quality of aspirated oocytes was also evaluated in relation to the in vitro maturation (IVM) outcome. In various BCS groups (BCS 1, 2, 3, 4), Kubovičová et al. (2012) observed a higher proportion of maturable oocytes (number of IVM-grade oocytes compared to the total number of collected oocytes; %) in cows with an optimal body condition than in animals tending to obesity or emaciation. Specifically, the proportion of maturable oocytes was 45.27 %, 48.97 %, 61.27 % and 35.48 % for BCS 1, 2, 3 and 4, respectively. Similarly, comparing the proportion of total mature and suitable oocytes for in vitro fertilization (IVF), a higher proportion of oocytes was detected in cows with an optimal body condition (79.85 %, 78.06 %, 96.13 % and 63.63 %, respectively). However, these differences were not statistically significant (Kubovičová et al., 2012).

Another study demonstrated that Holstein cows with an optimal body condition (BCS 3) had a higher quality of aspirated oocytes (oocytes selected for IVM compared to total recovered oocytes; %) than animals tending to emaciation (Chrenek et al., 2015). The results in the groups BCS 1, 2 and 3 were arranged as follows: 43.60 %, 57.60 % and 60.90 %, respectively. The differences compared to the BCS 1 group were statistically significant. This study also performed the evaluation according to actin grades. Actin cytoskeleton was classified into three quality grades (grade I – the best quality). There were no differences found among body condition groups (BCS 1, 2 and 3) in the proportion of embryos with grade I actin (41.67 %, 53.12 % and 42.86 %). However, summer months proved to be an important factor in the actin cytoskeleton quality (75.00 % of embryos with grade I actin) compared to the autumn or spring season (35.56 % or 28.57 %, respectively) (Chrenek et al., 2015).

The relationship between the body condition and quality of oocytes was also studied by Ruiz et al. (1996). The authors detected a higher proportion of cleaved embryos in cows with an optimal body condition than in animals tending to emaciation. Specifically, the proportion of cleaved embryos in the studied BCS groups (BCS 1, 2 and 3) was 49.1 %, 45.6 % and 61.1 %, respectively. Snijders et al. (2000) also found that better oocyte quality was associated with the optimal body condition of cows, particularly in cleavage rates and blastocyst formation. The authors state that in cows with BCS 1.5–2.5, the average number of collected oocytes was 7.0±1.8 vs. 8.5±1.48 in the BCS 3.3–4.0 group; the cleavage rate was 61.9 % vs. 75.7 % and the blastocyst rate (cultured from oocytes) 3.0 % vs. 9.9 %, respectively (Snijders et al., 2000).

The presented studies demonstrate a significant influence of the body condition of cows on their ovarian activity. The abovementioned literary sources are mainly focused on Holstein cattle, but the relationship between the body condition and reproduction traits has also been studied in other breeds, including local breeds. Thus, Fihri et al. (2005) studied the relationship between BCS and ovarian activity in different breeds in Morocco (Oulmès-Zaër breed, Holstein, crossbreeds). They reported a lower number of follicles per ovary and lower oocyte quality in animals with a lower body condition (BCS 2). Particularly, the average number of follicles, 22.98 (17.30, 23.80 and 37.83), was counted in cows with BCS 2, 3 and 5 (overall mean), and the average number of good-quality oocytes was 2.03 (1.07, 2.39 and 4.30, respectively) (Fihri et al., 2005).

Similarly, Domínguez (1995) studied the effect of animal body condition on the follicle number and oocyte quality in 449 cows (zebu, European breed, crossbred). Significant differences were found in the oocyte quality. In cows with BCS 1, 3 and 5, 16 %, 40 % and 42 % of proper-quality oocytes were found, respectively (Domínguez, 1995).

## 6 Conclusion

This review shows a close relationship between the cow body condition and reproduction traits not only from the viewpoint of animal husbandry parameters (e.g., 56 d nonreturn rate at first insemination, interval to first services, etc.), but also in the relationship to ovarian activity (e.g., oocyte recovery and in vitro embryo production). In general, most authors agree that the worsening of the body condition towards under-condition or over-condition is associated with worse results in embryo transfer (number and quality of embryos) and in vitro procedures (number and quality of aspirated oocytes). Therefore, it is necessary to pay better attention to the body condition of animals because this is an important factor affecting reproduction at the ovarian level.

## Data Availability

No data sets were used in this article.

## References

[bib1.bib1] (2018). Body condition estimation on cows from depht images using Convolutional Neural Networks. Comput. Electron. Agr..

[bib1.bib2] Aungier SPM, Roche JF, Diskin MG, Crowe MA (2014). Risk factors that affect reproductive target achievement in fertile dairy cows. J Dairy Sci.

[bib1.bib3] Azzaro G, Caccamo M, Ferguson JD, Battiato S, Farinella GM, Guarnera GC, Puglisi G, Petriglieri R, Licitra G (2011). Objective estimation of body condition score by modeling cow body shape from digital images. J Dairy Sci.

[bib1.bib4] Banos G, Brotherstone S, Coffey MP (2007). Prenatal Maternal Effects on Body Condition Score, Female Fertility, and Milk Yield of Dairy Cows. J Dairy Sci.

[bib1.bib5] Bastin C., Loker S, Gengler N, Sewalem A, Miglior F (2010). Genetic relationships between body condition score and reproduction traits in Canadian Holstein and Ayrshire first-parity cows. J Dairy Sci.

[bib1.bib6] Beam SW, Butler WR (1997). Energy Balance and Ovarian Follicle Development Prior to the First Ovulation Postpartum in Dairy Cows Receiving Three Levels of Dietary Fat. Biol Reprod.

[bib1.bib7] Bercovich A, Edan Y, Alchanatis V, Moallem U, Parmet Y, Honig H, Maltz E, Antler A, Halachmi I (2013). Development of an automatic cow body condition scoring using body shape signature and Fourier descriptors. J Dairy Sci.

[bib1.bib8] Berry DP, Buckley F, Dillon P, Evans RD, Rath M, Veerkamp RF (2003). Genetic parameters for body condition score, body weight, milk yield, and fertility estimated using random regression models. J Dairy Sci.

[bib1.bib9] Berry DP, Lee JM, MacDonald KA, Stafford K, Matthews L, Roche JR (2007). Associations Among Body Condition Score, Body Weight, Somatic Cell Count, and Clinical Mastitis in Seasonally Calving Dairy Cattle. J Dairy Sci.

[bib1.bib10] Berry DP, Lee JM, Macdonald KA, Roche JR (2007). Body Condition Score and Body Weight Effects on Dystocia and Stillbirths and Consequent Effects on Post-calving Performance. J Dairy Sci.

[bib1.bib11] Bewley JM, Peacock AM, Lewis O, Boyce RE, Roberts DJ, Coffey MP, Kenyon SJ, Schutz MM (2008). Potential for estimation of body condition scores in dairy cattle from digital images. J Dairy Sci.

[bib1.bib12] Bezdíček J, Louda F (2016). Reprodukční fyziologie skotu (Reproduction physiology in the cattle breeding). Cattle Research.

[bib1.bib13] Bezdíček J, Makarevič A, Stádník L, Kubovičová E, Louda F, Hegedüšová Z, Holásek R, Ducháček J, Stupka R (2015). Analysis of factors affecting the quantity and quality of embryo production in superovulated cows. Züchtungskunde.

[bib1.bib14] Boland MP, Lonergan P, O'Callaghan D (2001). Effect of nutrition on endocrine parameters, ovarian physiology, and oocyte and embryo development. Theriogenology.

[bib1.bib15] Buckley F, O'Sullivan K, Mee JF, Evans DR, Dillon P (2003). Relationships Among Milk Yield, Body Condition, Cow Weight, and Reproduction in Spring-Calved Holstein-Friesians. J Dairy Sci.

[bib1.bib16] Cam MA, Garipoglu AV, Kirikci K (2018). Body condition status at mating affects gestation length, offspring yield and return rate in ewes. Arch Anim Breed.

[bib1.bib17] Carvalho PD, Souza AH, Amundson MC, Hackbart KS, Fuenzalida MJ, Herlihy MM, Ayres H, Dresch AR, Vieira LM, Guenther JN, Grummer RR, Fricke PM (2014). Relationships between fertility and postpartum changes in body condition and body weight in lactating dairy cows. J Dairy Sci.

[bib1.bib18] Chebel RC, Mendonça LGD, Baruselli PS (2018). Association between body condition score change during the dry period and postpartum health and performance. J Dairy Sci.

[bib1.bib19] Chrenek P, Kubovičová E, Olexíková L, Makarevich AV, Toporcerová S, Ostró A (2015). Effect of body condition and season on yield and quality of *in vitro* produced bovine embryos. Zygote.

[bib1.bib20] Dechow CD, Rogers GW, Clay JS (2001). Heritabilities and correlations among body condition scores, production traits, and reproductive performance. J Dairy Sci.

[bib1.bib21] Dechow CD, Rogers GW, Clay JS (2002). Heritability and correlations among body condition score loss, body condition score, production and reproductive performance. J Dairy Sci.

[bib1.bib22] Domecq JJ, Skidmore AL, Lloyd JW, Kaneene JB (1997). Relationship between body condition scores and conception at first artificial insemination in a large dairy herd of high yielding Holstein cows. J Dairy Sci.

[bib1.bib23] Domínguez MM (1995). Effect of body condition, reproductive status and breed on follicular population and oocyte quality in cows. Theriogenology.

[bib1.bib24] Edmonson AJ, Lean IJ, Weaver LD, Farver T, Webster G (1989). A body Condition Scoring Chart for Holstein Dairy Cows. J Dairy Sci.

[bib1.bib25] Ferguson JD, Galligan DT, Thomsen N (1994). Principal descriptors of body condition score in holstein cows. J Dairy Sci.

[bib1.bib26] Ferguson JD, Azzaro G, Licitra G (2006). Body condition assessment using digital images. J Dairy Sci.

[bib1.bib27] Fihri FA, Klakhdissi H, Derqaoui H, Hajji Kh, Naciri M, Goumari A (2005). Genetic and nongenetic effects on the number of ovarian follicles and oocyte yield and quality in the bovine local (Oulmes Zaer), exotic breeds and their crosses in Morocco. Afr J Biotechnol.

[bib1.bib28] Friggens NC, Ingvartsen KL, Emmans GC (2004). Prediction of Body Lipid Change in Pregnancy and Lactation. J Dairy Sci.

[bib1.bib29] Gillund P, Reksen O, Grohn YT, Karlberg K (2001). Body condition related to ketosis and reproductive performance in Norwegian dairy cows. J Dairy Sci.

[bib1.bib30] Gruber L, Ledinek M, Steininger F, Fuerst-Waltl B, Zottl K, Royer M, Krimberger K, Mayerhofer M, Egger-Danner C (2018). Body weight prediction using body size measurements in Fleckvieh, Holstein, and Brown Swiss dairy cows in lactation and dry periods. Arch Anim Breed.

[bib1.bib31] Halachmi I, Polak P, Roberts DJ, Klopcic M (2008). Cow Body Shape and Automation of Condition Scoring. J Dairy Sci.

[bib1.bib32] Hansen MF, Smith ML, Smith LN, Jabbar KA, Forbes D (2018). Automated monitoring of dairy cow body condition, mobility and weight using a single 3D video capture device. Comput Ind.

[bib1.bib33] Kadokawa H, Tameoka N, Uchiza M, Kimura Y, Yonai MA (2008). Field Study on the Relationship Between Body Condition and Embryo Production in Superovulated Holstein Yearling Heifers. J Dairy Sci.

[bib1.bib34] Koenen EPC, Veerkamp RF, Dobbelaar P, De Jong G (2001). Genetic analysis of body condition score of lactating Dutch Holstein and Red-and-White heifers. J Dairy Sci.

[bib1.bib35] Kubovičová E, Makarevič A, Hegedušová Z, Slezáková M, Bezdíček J (2012). Effect of body condition score on oocyte yield and in vitro embryo development. Cattle Research.

[bib1.bib36] Kuźnicka E, Rant W, Radzik-Rant A, Kunowska-Slósarz M, Balcerak M (2016). The ovulation rate, plasma progesterone and estradiol concentration, and litter size of a local ewe breed kept in a barn vs. those kept under an overhead shelter. Arch Anim Breed.

[bib1.bib37] Ledinek M, Gruber L, Steininger F, Fuerst-Waltl B, Zottl K, Royer M, Krimberger K, Mayerhofer M, Egger-Danner C. (2019). Analysis of lactating cows on commercial Austrian dairy farms: the influence of genotype and body weight on efficiency parameters. Arch Anim Breed.

[bib1.bib38] Loker S, Miglior F, Koeck A, Neuenschwander TFO, Bastin C, Jamrozik J, Schaeffer LR (2012). Relationship between body condition score and health traits in first-lactation Canadian Holsteins. J Dairy Sci.

[bib1.bib39] Makarevich AV, Pivko J, Kubovičová E, Hegedušová Z, Louda F (2011). Ovarian follicle atresia in dairy cows in relation to the body condition. Journal of Microbiology, Biotechnology and Food Sciences.

[bib1.bib40] Makarevich AV, Kubovičová E, Hegedüšová Z, Bezdíček J, Louda F, Pivko J (2012). Impact of body condition of
dairy cows on ovarian state and oocyte quality. Book of Abstracts and poster
presentation.

[bib1.bib41] Makarevich A, Kubovičová E, Olexiková L, Hegedušová Z, Chrenek P (2012). Influence of body condition of dairy cows on the oocyte and embryo quality in vitro. Reproduction in Domestic Animals, 47.

[bib1.bib42] Makarevich AV, Špaleková E, Stádník L, Bezdíček J, Kubovičová E (2018). Functional characteristics of bovine spermatozoa in relation to the body condition score of bulls. Slovak Journal of Animal Science (SJAS).

[bib1.bib43] Murphy MG, Enright WJ, Crowe MA, McConnell K, Spicer LJ, Boland MP, Roche JF (1991). Effect of dietary intake on pattern of growth of dominant follicles during the oestrous cycle in beef heifers. J Reprod Fertil.

[bib1.bib44] Nowak TA, Jaškowski JM, Szczepankiewicz D, Olechnowicz J (2011). Quality and number of cumulus–oocyte complexes (COC) and concentrations of leptin and ghrelin in blood and follicular fluid depending on the body condition of cows. Medycyna Weterynaryjna.

[bib1.bib45] Oba M, Miyashita S, Nishii R, Koiwa M, Koyama H, Ambrose DJ, Dochi O (2013). Short communication: Effects of serum obtained from dairy cows with low or high body condition score on *in vitro* embryo development. J Dairy Sci.

[bib1.bib46] Pivko J, Makarevich AV, Kubovičová E, Ostro A, Hegedusova Z, Louda F (2012). Histopathological alterations in the antral ovarian follicels in dairy cows with a tendency to emaciation. Histol Histopathol.

[bib1.bib47] Pryce JE, Coffey MP, Simm G (2001). The Relationship Between Body Condition Score and Reproductive Performance. J Dairy Sci.

[bib1.bib48] Pryce JE, Coffey MP, Brotherstone SH, Woolliams JA (2002). Genetic relationships between calving interval and body condition score conditional on milk yield. J Dairy Sci.

[bib1.bib49] Ptáček M, Ducháček J, Stádník L, Hakl J, Fantová M (2017). Analysis of multivariate relations among birth weight, survivability traits, growth performance, and some important factors in Suffolk lambs. Arch Anim Breed.

[bib1.bib50] Qu Y, Fadden AN, Traber MG, Bobe G (2014). Potential risk indicators of retained placenta and other diseases in multiparous cows. J Dairy Sci.

[bib1.bib51] Roche JR, Lee JM, MacDonald KA, Berry DP (2007). Relationships among body condition score, body weight, and milk production variables in pasture-based dairy cows. J Dairy Sci.

[bib1.bib52] Ruiz LL, Alvarez N, Nunez I, Montes I, Solano R, Fuentes D, Pedroso R, Palma GA, Brem G (1996). Effect of body condition on the developmental competence of IVM/IVF bovine oocytes. Theriogenology.

[bib1.bib53] Shelley AN, Lau DL, Stone AE, Bewley JM (2016). Short communication: Measuring feed volume and weight by machine vision. J Dairy Sci.

[bib1.bib54] Shigeta M, Ike R, Takemura H, Ohwada H (2018). Automatic Measurement and Determination of Body Condition Score of Cows Based on 3D Images Using CNN. Journal of Robotics and Mechatronics.

[bib1.bib55] Silke V, Diskin MG, Kenny DA, Boland MP, Dillon P, Mee JF, Sreenan JM (2002). Extent, pattern and factors associated with late embryonic loss in dairy cows. Anim Reprod Sci.

[bib1.bib56] Sirotkin AV, Makarevich AV, Makovicky P, Kubovicova E (2013). Ovarian, metabolic and endocrine indexes in dairy cows with different body condition scores. J Anim Feed Sci.

[bib1.bib57] Snijders SE, Dillon P, O'Callaghan D, Boland MP (2000). Effect of genetic merit, milk yield, body condition and lactation number on *in vitro* oocyte development in dairy cows. Theriogenology.

[bib1.bib58] Spoliansky R, Edan Y, Parmet Y, Halachmi I (2016). Development of automatic body condition scoring using a low-cost 3-dimensional Kinect camera. J Dairy Sci.

[bib1.bib59] Tančin V, Mikláš Š, Mačuhová L. (2018). Possible physiological and environmental factors affecting milk production and udder health of dairy cows: A review. Slovak J Anim Sci.

[bib1.bib60] Tiezzi F, Maltecca C, Cecchinato A, Penasa M, Bittante G (2013). Thin and fat cows, and the nonlinear genetic relationship between body condition score and fertility. J Dairy Sci.

[bib1.bib61] Weber A, Salau J, Haas JH, Junge W, Bauer U, Harms J, Suhr O, Schönrock K, Rothfuß H, Bieletzki S (2014). Estimation of backfat thickness using extracted traits from an automatic 3D optical system in lactating Holstein-Friesian cows. Livest Sci.

[bib1.bib62] Wildman EE, Jones GM, Wagner PE, Boman RL, Troutt HF, Lesch TN (1982). A dairy cow body condition scoring system and its relationship to selected production characteristics. J Dairy Sci.

